# Monolithic integration of SiC diode detectors into scanning X-ray microscopy optical elements for *in situ* beam diagnostics

**DOI:** 10.1107/S1600577526007113

**Published:** 2026-08-05

**Authors:** Gabriele Trovato, Simone Finizio, Samuele Moscato, Jörg Raabe, Joakim Reuteler, Massimo Camarda

**Affiliations:** a Università degli Studi di Catania, Dipartimento di Fisica e Astronomia ‘Ettore Majorana’, Via Santa Sofia 64, 95123Catania, Italy; b STLab srl, Via Anapo 53, 95126Catania, Italy; c Istituto Nazionale di Fisica Nucleare – INFN Sezione di Catania, Via S. Sofia 64 95123Catania Italy; d Istituto per la Microelettronica e Microsistemi CNR-IMM Sezione di Catania, Strada VIII Zona Industriale 5 95121Catania Italy; e Swiss Light Source Paul Scherrer Institut 5232Villigen PSI Switzerland; f Università degli Studi di Catania, Dipartimento di Ingegneria Elettrica, Elettronica e Informatica (DIEEI), Viale Andrea Doria 6, I-95125Catania, Italy; g ScopeM ETH Zürich 8093Zürich Switzerland; h SenSiC GmbH, DeliveryLAB, 5232Villigen PSI, Switzerland; European XFEL, Germany

**Keywords:** silicon carbide (SiC), scanning transmission X-ray microscopy (STXM), beam diagnostics, normalization of microspectroscopy data

## Abstract

Reliable monitoring of the incident X-ray beam intensity and position is essential in soft X-ray microspectroscopy, where beam instabilities directly affect data normalization and achievable spatial resolution. Conventional beam diagnostics are often implemented using offline detectors located far from the sample or by measuring downstream on empty regions of the sample support membrane, not fully reflecting the experimental conditions experienced during measurements. In this work, the development of functionalized soft X-ray optical elements based on monolithic silicon carbide (SiC) diode detectors, enabling real-time *in situ* beam diagnostics directly within a scanning transmission X-ray microscopy (STXM) setup, is presented. Two complementary device geometries are investigated: order-sorting aperture (OSA)-based detectors and center-stop-based detectors. The devices are fabricated using plasma-focused-ion-beam milling and substrate thinning, allowing the integration of optical and sensing functionalities with minimal changes to the beamline and endstation setup. The performance of the functionalized optics is experimentally validated at the PolLux STXM beamline of the Swiss Light Source. The devices provide simultaneous access to the transverse beam position and the incident beam intensity *I*_0_ through the unfocused 0th-order component. Using this component, the OSA detector enabled simultaneous measurements of the intensity and transverse displacement of the beam from the focused beam illuminating the sample. Between 700 and 1000 eV, the SiC measured current exhibited linear correlation to the avalanche photodiode (APD) signal, with coefficient of determination *R*^2^ = 0.997. Position-sensitive detection was possible with a four-sector diode, where all sectors were electrically continuous and beam responsive, while gaps between the quadrants were insensitive. These results demonstrate the feasibility of SiC-based functionalized X-ray optics as beam diagnostic tools for soft X-ray microscopy and spectroscopy.

## Abstract 2

Silicon carbide detectors are monolithically integrated into order-sorting apertures and center stops for *in situ* soft X-ray beam diagnostics in STXM. Functionalized components enable simultaneous monitoring of incident beam intensity and the transverse position at sample-relevant locations without degrading optical performance.

## 1. Introduction

X-ray microspectroscopy at the soft X-ray energy range is a powerful technique for the investigation of chemical, electronic and magnetic properties of materials combining high spatial and energy resolutions (Kirz *et al.*, 1995 ▸). Amongst the portfolio of X-ray microspectroscopy techniques, scanning transmission X-ray microscopy (STXM), also known as nanofocus imaging, has become a widely adopted tool at synchrotron radiation facilities for element-specific and dichroic microspectroscopy in fields ranging from condensed matter physics to materials science and environmental and life sciences (Watts *et al.*, 2006 ▸; Jacobsen, 1999 ▸; Liu *et al.*, 2014 ▸).

Recent advancements have further expanded the application of STXM from chemically sensitive nano-imaging to multimodal spectromicroscopy systems. Many soft X-ray STXM endstations are now equipped with state-of-the-art scanning controls, detection techniques, cryogenic and *in situ* sample environment, tomography, and time-resolved acquisitions for varied applications in material sciences, magnetic phenomena, energy materials, environmental research and biological research (Feggeler *et al.*, 2023 ▸).

**Figure 1:**
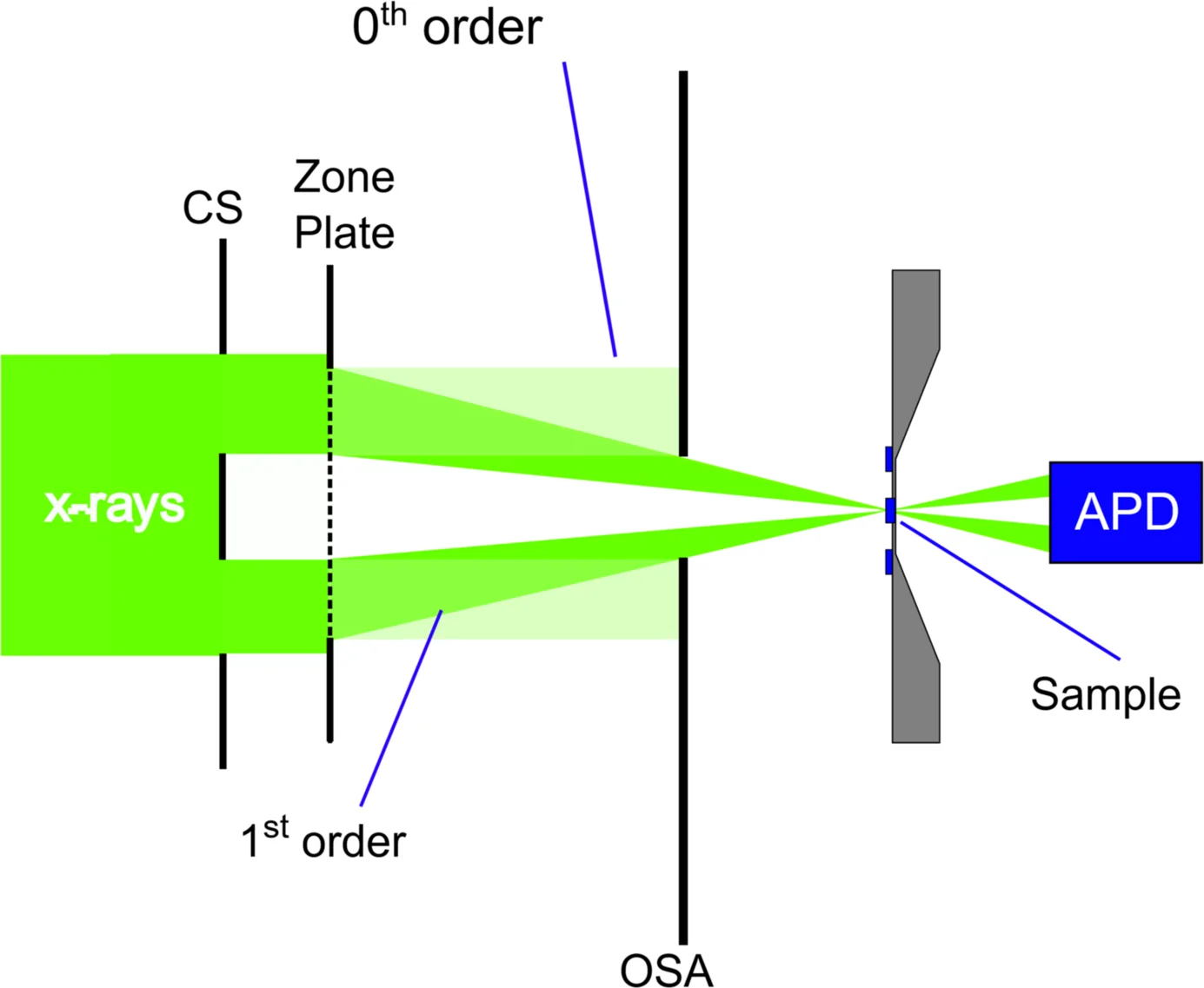
Sketch of the STXM setup, composed of a center stop, a Fresnel zone plate, an order-sorting aperture and an SiN membrane used as a support for the sample. Behind the membrane, an avalanche photodiode detector is used to monitor the transmitted photon flux.

The operating principle of STXM (Raabe *et al.*, 2008 ▸), sketched in Fig. 1 ▸, consists of using a diffractive optical element, a Fresnel zone plate (FZP), to focus a monochromatic X-ray beam onto a nanometric spot on an X-ray transparent sample. The intensity of the X-ray beam transmitted across the sample is then recorded by a point detector, which is normally either a photomultiplier tube combined with a phosphor screen that converts the X-ray photons into visible-light photons, typically used at the lower energy ranges (200–500 eV), or an avalanche photodiode (APD) for direct X-ray photon detection at higher energies (500 eV to 1.5 keV). An image is formed by scanning the sample with a piezoelectric stage and recording the transmitted X-ray beam intensity for each pixel of the image. The achievable spatial resolutions in the soft X-ray energy range are typically of 20 nm, with the current state-of-the-art being around 7 nm, limited by lithographical fabrication challenges for the higher-resolution FZPs (Rösner *et al.*, 2020 ▸). As soft X-ray FZPs are diffractive optics with a focusing efficiency typically of about 10%, to guarantee that only the focused light reaches the sample, a combination of a center stop (CS) and order-sorting aperture (OSA) is used to block both the unfocused light and the higher focal orders from the FZP (Tong *et al.*, 2023 ▸).

In addition to advances in diffractive optics, coherent scanning techniques have gained importance for the STXM community, emphasizing the effect of instrumentation in quantitative zone-plate-based X-ray spectromicroscopy, which enables an increase in the accuracy of chemical information at the nanometre scale for heterogeneous samples (Marcus *et al.*, 2021 ▸). The spectral distortions due to the energy and spatially varying point spread function of standard STXM can be a source of unreliability in nanochemical analysis especially when the heterogeneous elements of the samples are near the size of the probe. This reflects the general tendency towards quantitative measurements with STXM that requires the instrumental stability and robust normalization.

Both the quality and reproducibility of STXM imaging experiments critically depend on the stability, position and intensity of the incident X-ray beam, as transmission data are referenced to the incident beam intensity, usually referred to as the *I*_0_ signal. Variations of beam intensity and transverse position can introduce systematic artifacts affecting both quantitative analysis and limiting sensitivity, especially for low signal-to-noise measurements (Watts *et al.*, 2022 ▸). Intensity fluctuations impact the normalization of absorption spectra, while beam drifts and vibrations can lead to distortions and blurring in spatially resolved measurements. For these reasons, a reliable *I*_0_ monitoring is a key requirement for modern soft X-ray microscopy beamlines (Watts *et al.*, 2006 ▸; Watts *et al.*, 2018 ▸). Some examples of both the effect of unstable beam conditions and of the importance of a proper *I*_0_ normalization on the quality of acquired images and spectra are shown in the supplementary information. The current state-of-the-art approach for normalizing microspectroscopic data to the *I*_0_ signal consists of acquiring data from the sample of interest and a neighboring empty region on the supporting SiN membrane for each energy point of the acquired spectrum. The data from the sample region are then divided by the *I*_0_ signal from the neighboring region of the sample (Watts *et al.*, 2022 ▸). However, this method is both time-consuming, as it involves the acquisition of a non-scientifically relevant region of the sample for each energy point, and cannot normalize for variations of the beam intensity occurring in timescales comparable to the total acquisition time within one energy point. In addition, the choice of regions of interest on the sample is limited to places close to an empty membrane region, reducing the ensemble of systems that can be investigated.

Synchronized incident beam monitoring in STXM is gaining importance, and recent applications have demonstrated that real-time beam-intensity monitoring can correct intensity variations associated with synchrotron top-up injection in STXM images (Jiao *et al.*, 2025 ▸). Incident beam stability is then not only a performance parameter, but directly affects image contrast, spectral normalization and reliability in long-duration measurements.

The ideal scenario for data normalization would therefore be to have *liveI*_0_ monitoring during the entire image acquisition and implementations of this solution should be, ideally, transparent to the existing beamline/endstation setups and not impact the photon flux delivered to the sample.

Silicon carbide (SiC) is a particularly suitable material for this purpose due to its wide bandgap, radiation hardness, low dark current, and chemical and mechanical stability (De Napoli, 2022 ▸). SiC-based detectors have been extensively explored as beam diagnostic systems (Trovato *et al.*, 2025*b* ▸). In the case of synchrotron radiation facilities at hard and tender X-ray energies, free-standing SiC membranes are an attractive solution for beam monitoring due to their robustness and low absorption losses (Trovato *et al.*, 2025*b* ▸; Medina *et al.*, 2025 ▸). The extension of freestanding SiC membranes for detector technology applications to the soft X-ray regime was explored (Trovato *et al.*, 2025*a* ▸). However, even though SiC free-standing membranes can be realized with sub-100 nm thicknesses, due to the lower attenuation length of soft X-rays compared to tender and hard X-ray radiation (Henke *et al.*, 1993 ▸), a transmission-type sensor will negatively impact the beam intensity delivered to the sample.

A possible strategy to achieve these goals is to integrate beam intensity and position diagnostics directly onto the existing X-ray optical elements, and making use of ‘discarded’ parts of the X-ray beam, *i.e.* the unfocused part of the beam. In particular, we embed position- and intensity-sensitive detectors into the OSA and CS elements, enabling us to perform *in situ* beam diagnostics under conditions as close as possible to those experienced by the sample, requiring only minor modifications of the endstation setup. We make use of the ‘discarded’ part of the X-ray beam, *i.e.* the unfocused 0th-order light from the FZP, enabling us to provide a live *I*_0_ monitoring without affecting the beam intensity delivered to the sample. The relation between the focused first-order beam delivered to the sample and the unfocused 0th-order beam-induced current is possible to obtain as a calibration curve, with dependence on the photon energy, on the beamline and on the FZP focusing efficiency.

In this work, we present the design, fabrication and proof-of-concept experimental validation of functionalized soft X-ray optical elements based on monolithic SiC diode detectors. Two complementary device geometries are investigated: OSA-based and CS-based detectors. Both devices were fabricated using plasma focused-ion-beam (PFIB) milling and were characterized under routine imaging conditions at the PolLux endstation of the Swiss Light Source (SLS) (Raabe *et al.*, 2008 ▸). The functionalized optics enable the simultaneous live measurement of the *I*_0_ signal and transverse position.

## 2. Materials and methods

### 2.1. Devices fabrication

The devices were fabricated on a 15 mm × 15 mm SiC chip. On the frontside of the chip, a 100 nm-thick Al layer was deposited with an EVATEC BAK-UNI E-beam evaporator (EVATEC AG, https://evatecnet.com) and lithographically patterned into four-sector geometries with a total active area of 2 mm × 2 mm, using a DWL 66+ Laser Lithography System (Heidelberg Instruments Mikrotechnik GmbH, https://heidelberg-instruments.com).

**Figure 2:**
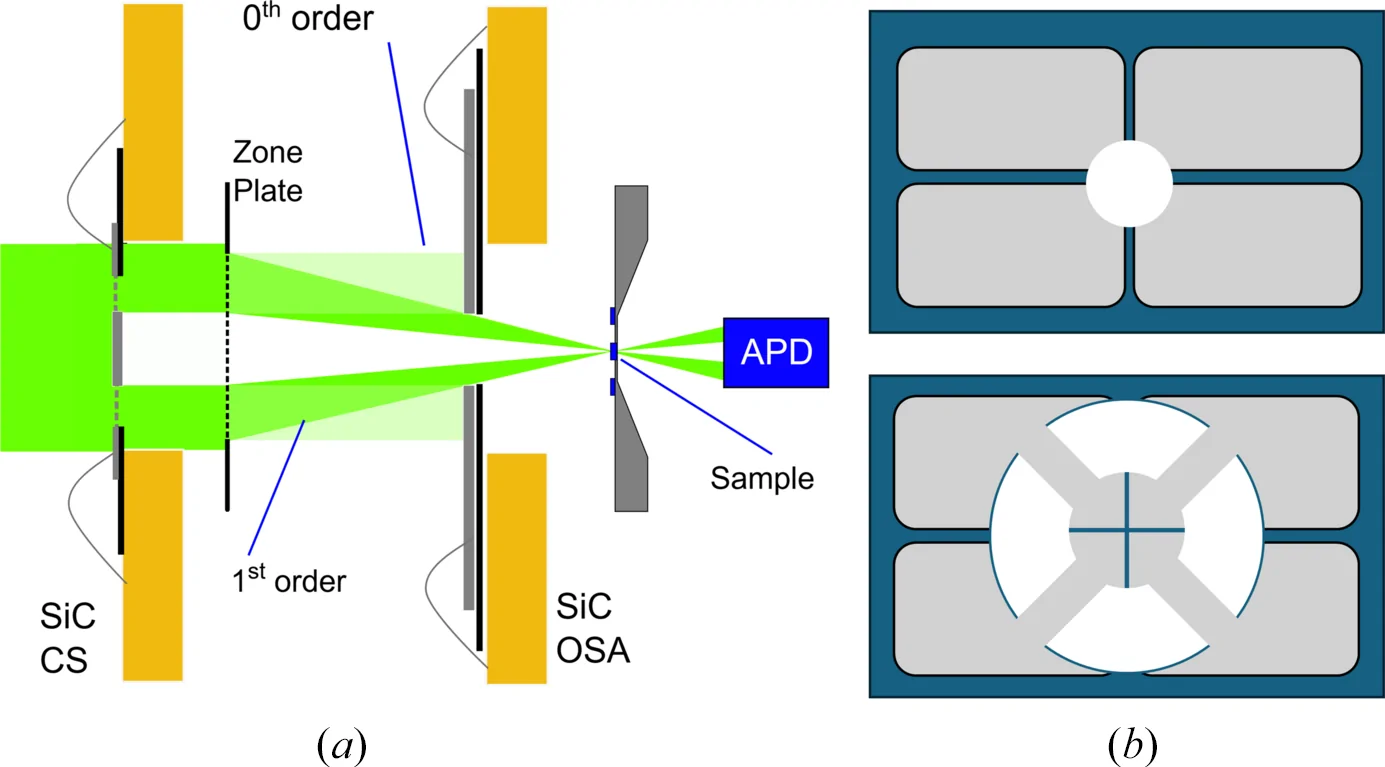
(*a*) Complete setup for STXM measurement with the substitution of the standard elements with the functionalized SiC center stop and OSA. (*b*) Schematic front view of the OSA (top) and CS (bottom).

The STXM implementation using the SiC functionalized optical elements, together with the front-view geometries of the OSA and CS devices, is shown in Fig. 2 ▸. At the soft X-ray energy range, typically used FZP optics have focal distances of a few mm, which requires a miniaturization of the OSA, as this needs to be positioned between the FZP and the sample. Especially at low energies and with high-resolution FZP, this can provide for challenging experimental conditions (Rösner *et al.*, 2020 ▸). This is therefore one of the boundary conditions for the functionalization of the OSA, with the requirement to be below 100 µm thickness. The other requirement is that the thickness of the device should be sufficiently high to completely block the X-ray beam in the unmilled regions. This requirement is already reached at thicknesses of a few micrometres [attenuation length of SiC at 1 keV is approximately 1.7 µm (Henke *et al.*, 1993 ▸)]. As standard SiC diode substrates are fabricated with a ∼0.4 mm thickness, mechanical thinning down to 0.1 mm in thickness is required prior to the fabrication of the OSA pinhole.

Following the mechanical thinning step, the functionalized OSAs were fabricated by opening pinholes with diameters varying from 50 to 100 µm with PFIB milling. PFIB milling was preferred over laser-beam cutting thanks to its ability to produce a well defined feature edge whilst minimizing damage to surrounding regions (see supplementary information for comparison between the two methods). PFIB milling was performed using a 2.5 µA/30 kV Xe ion beam in a Helios Hydra FIB/SEM instrument installed at the ScopeM laboratories of ETH Zürich.

As PFIB milling tends to follow a conical shape for thicker samples; in order to avoid damage to the top surface of the SiC diode around the OSA pinhole, the PFIB milling was performed from the backside. This detail is particularly important because the devices operate as Schottky diodes, in which the rectifying junction is formed at the metal–semiconductor contact. Milling from the backside of the chip avoids ablation and melting of the metal, reducing the chance of short circuits between the two sides of the junction.

**Figure 3:**
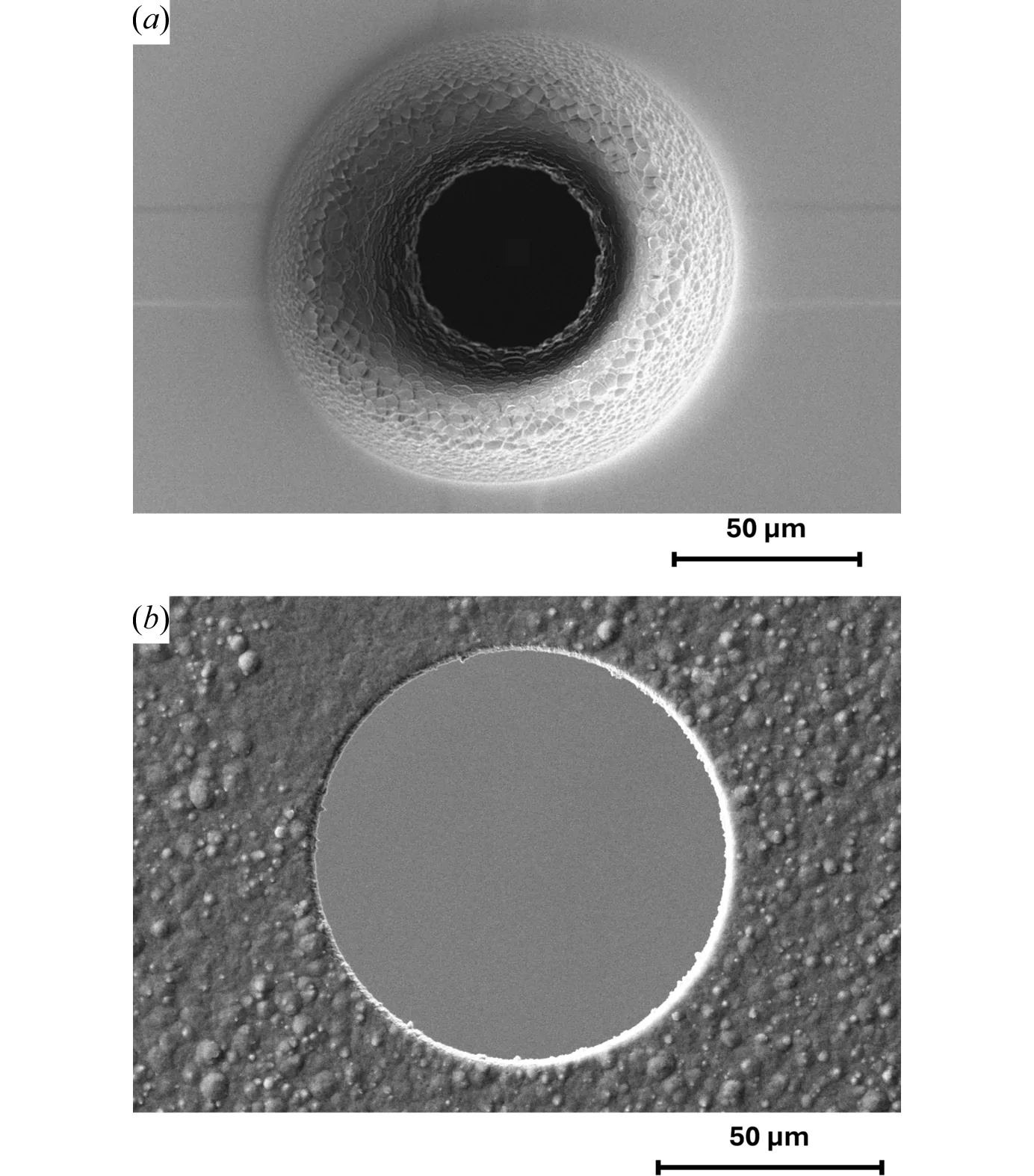
Scanning electron micrographs of an OSA fabricated out of a 100 m-thick SiC substrate when the PFIB process is realized from the frontside (*a*) and from the backside (*b*) of the device. The images are taken during the process from the frontside and after the process from the backside, respectively.

A comparison of the surface quality differences between frontside and backside milling processes is shown in Fig. 3 ▸, underlining the importance of backside milling to guarantee a high surface quality on the final functionalized OSA pinhole. The final diameter of the SiC OSA used in this work is of 90 µm, chosen to enable transmission of the focused first-order beam according to the alignment configuration at PolLux but block the unfocused and higher-order focuses of the FZP.

Electrical measurements performed with a Keithley 2450 source meter (Tektronix, https://www.tek.com) before and after the PFIB milling process confirmed that there are no changes in the diode response after the milling from the backside, independently of the parameters utilized, indicating that the method is robust for the processing of SiC devices.

**Figure 4:**
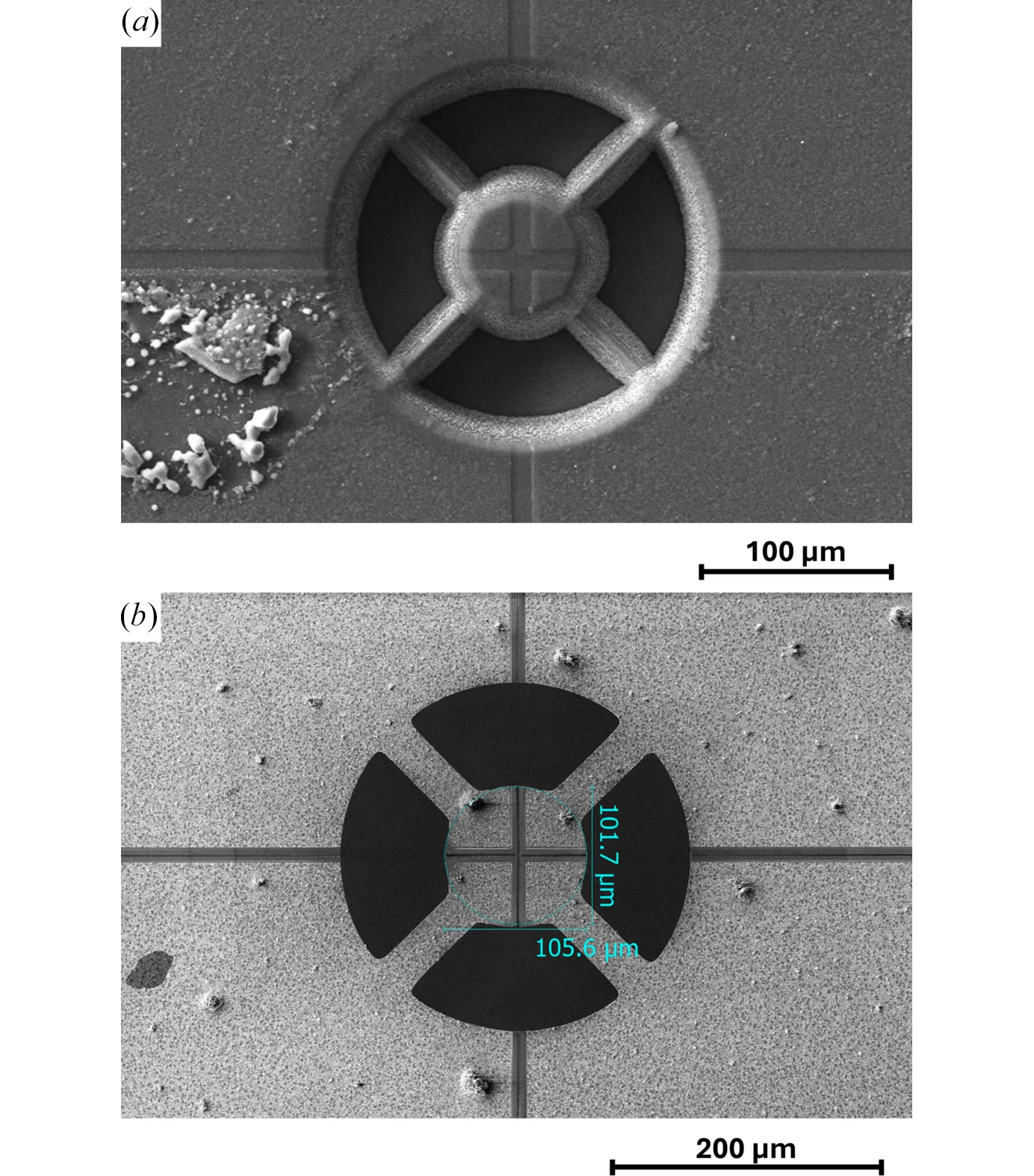
SEM images of center-stop structures patterned by PFIB lithography. (*a*) CS patterned from the frontside of the SiC substrate. The damage of the support arms, which caused the central disk to be electrically insulated from the rest of the diode, can be observed in the image. (*b*) CS patterned from the backside of the SiC substrate. The junction of the diode is not damaged in the support arms, allowing for a good electrical contact of the central disk with the rest of the diode.

The functionalized CSs were also fabricated with PFIB milling, using similar parameters to the ones employed for the fabrication of the functionalized OSAs described above. However, in this case, the CS geometry poses additional fabrication challenges, as the central region of the device needs to be electrically connected to the surrounding diode sectors. For this reason, frontside PFIB milling is even less suited for this device than for the fabrication of the OSA pinholes, as shown in Fig. 4 ▸(*a*): the conical shape of the milling produces an excessive thinning down of the support arms connecting the central region to the rest of the diode, which are also meant to provide an electrical contact.

Therefore, the CS devices were also fabricated through backside milling, as shown for one representative device in Fig. 4 ▸(*b*). With this fabrication method, the central region of the CS device could remain electrically connected to the rest of the diode.

Following the milling, the backside of the devices was metallized with a 100 nm-thick Al film. Up to now, multiple devices on the same 15 mm × 15 mm SiC chip were fabricated in parallel. The single functionalized devices, with a 3 mm × 3 mm footprint, were cut from the larger chip with a Disco DAD 341 dicing saw (DISCO Corporation, https://www.disco.co.jp/).

As for the OSA detector, the functionalized CS was fabricated with the same diameter as the equivalent, unfunctionalized, component utilized at the PolLux beamline (100 m). Both functionalized devices have been fabricated such that the sensing region of SiC is placed in optical regions which are otherwise non-transmissive of the focused first-order beam.

**Figure 5:**
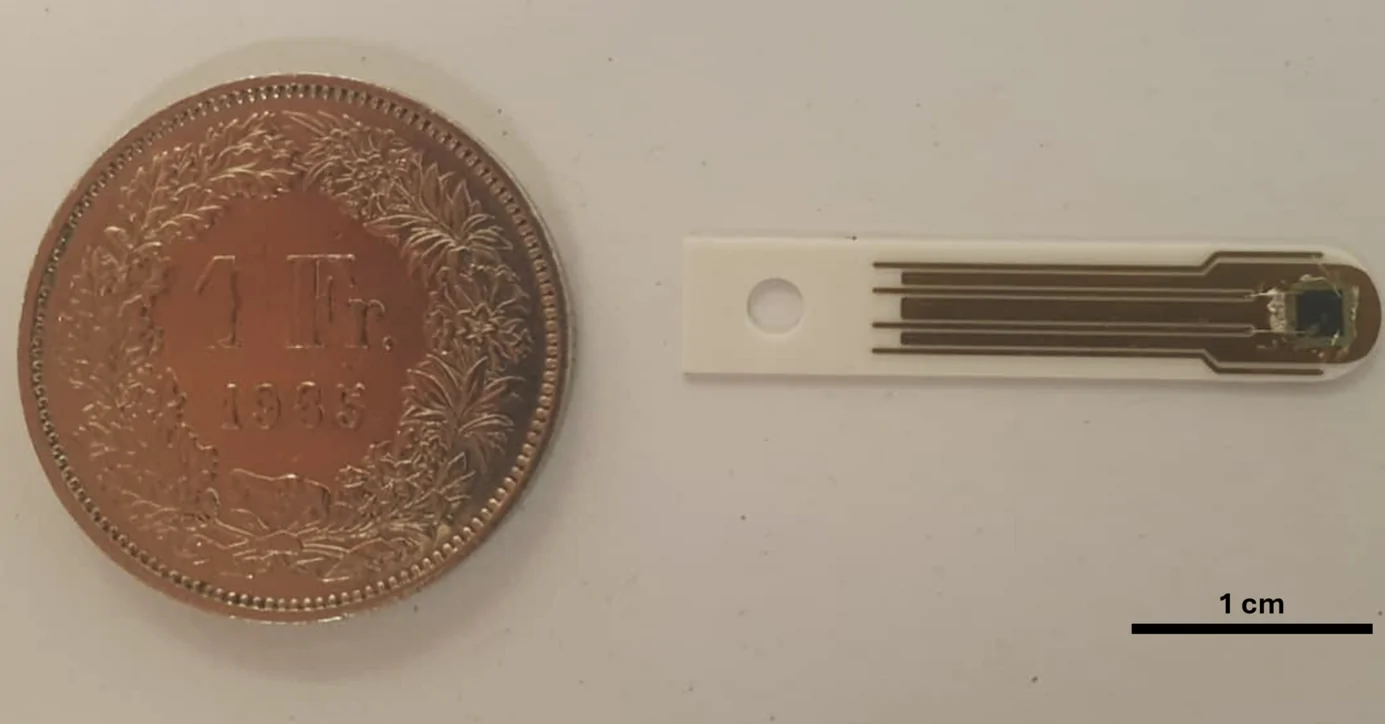
Image of the SiC OSA detector mounted on the 0.2 mm-thick Rogers RO4003C ceramic and compared with a 1 Swiss franc coin.

Finally, the finished devices were attached to custom-designed printed circuit boards (fabricated with a 0.2 mm-thick Rogers RO4003C ceramic, 35 µm-thick Au-coated Cu contacts) using a conducting Ag-based paste (RS 186-3600). The frontside of the diode was then contacted with 35 µm diameter Al wirebonds. A photograph of the device mounted on the circuit board is shown in Fig. 5 ▸.

### 2.2. Experimental setup

The proof-of-concept experiments were performed at the PolLux STXM endstation of the Swiss Light Source. The beamline operates in the soft X-ray energy range, from about 280 to 1500 eV, covering the elemental absorption edges of carbon, nitrogen, oxygen and sodium (*K*-edge), of potassium, iron, cobalt and nickel (*L*-edges), and of the rare earth elements (*M*-edges) (Raabe *et al.*, 2008 ▸). The source point is imaged onto a secondary source located 1 m behind the experimental endstation. The size of the secondary source can be controlled by a slit system, allowing one to control the degree of coherence of the beam and its energy resolution (Flechsig *et al.*, 2007 ▸), of particular importance both to achieve the best focusing conditions with the FZP (Rösner *et al.*, 2020 ▸) and for performing spectromicroscopy experiments.

For the experiments described here, we replaced the existing OSA and CS of the PolLux STXM endstation with the functionalized devices described above. With the exception of the installation of coaxial cables, no changes to the STXM setup (or to the beamline) were performed. The Au FZP used for this experiment has a 240 µm diameter, with 35 nm the outermost zone width and a thickness of the electroplated gold of 200 nm. The center stop element of the beamline has a 100 µm diameter, with a 2 µm-thick electrodeposited gold metallization.

The detector used for the experiments reported here was a Hamamatsu S12023-05 Si Avalanche Photodiode (Hamamatsu Photonics KK, https://www.hamamatsu.com/eu/en/product/optical-sensors/apd/si-apd/S12023-05.html), installed in a custom-designed Cu casing inside the vacuum chamber of the endstation with a 60 dB preamplifier unit as close as possible to the diode. The amplified APD signal was then collected, outside of the endstation, by a Hamamatsu C9744 photon counting unit, which then generated a total transistor logic signal used by the control system of the endstation to record a photon detection event.

The electrical readout of the SiC detectors was performed using the SenSiC PCR4 readout system (SenSiC, https://www.sensic.ch/products). The PCR4 is a low-noise picoammeter designed for the simultaneous amplification and measurement of currents in four independent channels. Each channel features a trans-impedance current amplification stage with adjustable measurement ranges from ± 25 nA to ± 50 mA, offering a 24-bit dynamic range and bipolar current readout. The PCR4 allows for acquisition rates of up to 10 kSa s^−1^, featuring an analog bandwidth of 800 Hz (−3 dB), which enables integration times ranging from the millisecond down to the sub-millisecond range, compatible with the typical pixel dwell times of the PolLux STXM endstation. A bipolar bias voltage source (± 20 V) for sensor polarization is also integrated. Each of the four sectors of the SiC diode was read out independently, allowing both for the simultaneous acquisition of the total photocurrent (*I*_0_) and of differential signals that correlate with the position of the beam illuminating the FZP. The readout system was integrated into the PolLux beamline control infrastructure via a local EPICS input/output controller. This allowed for the synchronization of the recorded *I*_0_ signal with the recorded photon count rates for each pixel in the STXM images.

## 3. Results and discussion

### 3.1. Experimental results

**Figure 6:**
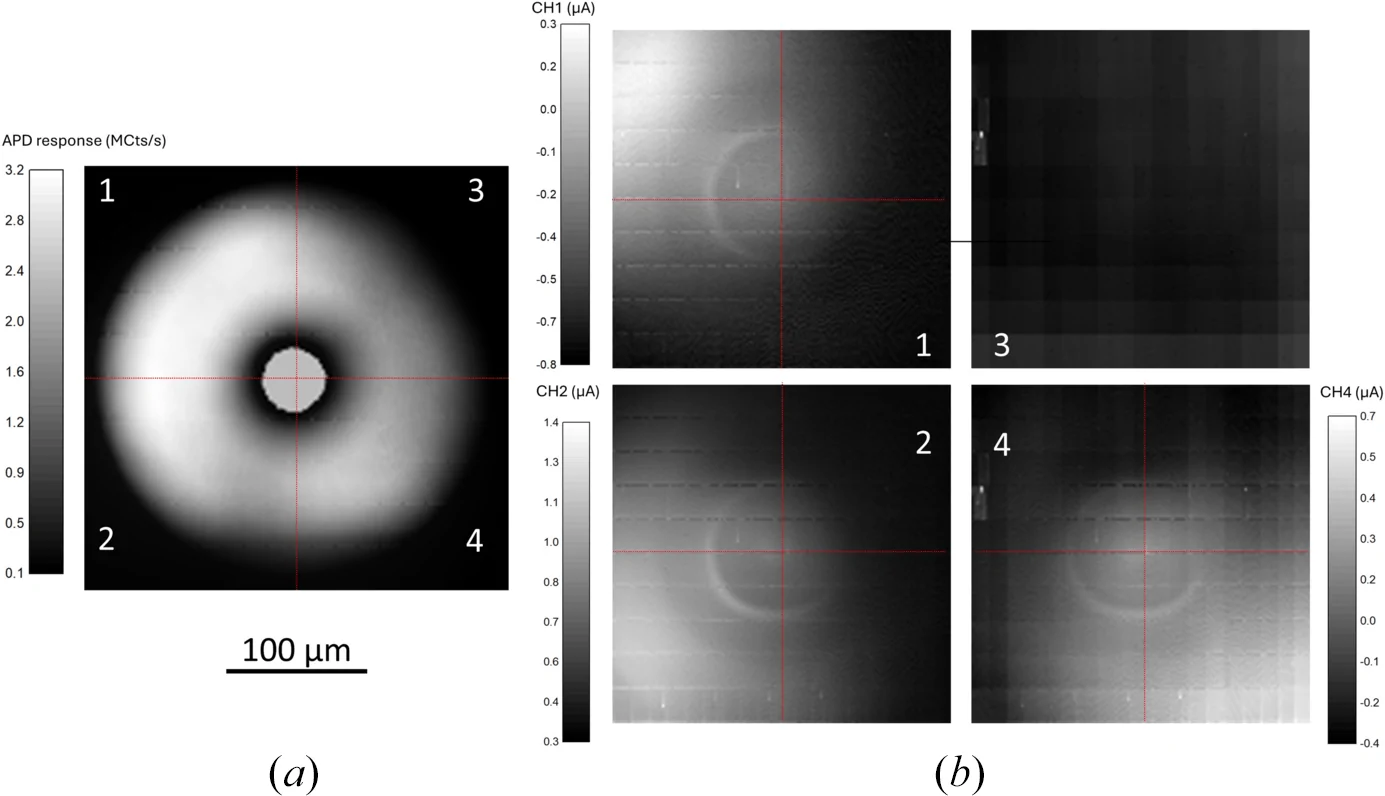
(*a*) STXM image of the X-ray beam illuminating the SiC OSA, where the zeroth-order focus (outer ring) and the first-order focus (central disk) can be observed. (*b*) Response of the four individual sectors of the SiC diode [marked in (*a*)] achieved by scanning the OSA across the beam defined by the Fresnel zone plate installed in the PolLux STXM. The response of each channel, as expected, is larger when the beam irradiates the corresponding diode sector. Sector 3 shows no response due to a faulty contact.

The first measurements were performed on the functionalized OSA. The first image, shown in Fig. 6 ▸(*a*), is a so-called OSA scan, where the OSA was scanned with a focused beam. This is a standard procedure performed upon installation of a new OSA, used to align it with the optical axis of the FZP. The electrical response of the four-sector SiC diode was recorded in parallel to the STXM measurements and is shown in Fig. 6 ▸(*b*). The measurements were performed with an X-ray beam energy of 1 keV. Note that due to a ripped-off wirebond on the SiC device, sector 3 did not exhibit any electrical response [see Fig. 6 ▸(*b*)] . Each functioning sector exhibits the expected variation in signal as a function of beam displacement, demonstrating the position sensitivity of the device. The differential response between opposite sectors allows one to infer the beam position in the plane of the OSA, while the sum of all sector signals provides the total 0th-order-induced photocurrent, translatable as the incident beam intensity *I*_0_ through the calibration curve.

It should be emphasized that this measurement provides a local and relative indicator of the beam spot-size changes at the position of the OSA. As the signal is collected in only one longitudinal section, shifts in the position of the beam cannot be distinguished from changes of the beam angle. The difference of responses of two sectors should thus be understood as the indication of the changes in the beam illumination of the OSA/FZP assembly, whereas the sum of responses of both sectors gives the total zeroth-order current, which is proportional to the incident beam intensity.

In order to determine if the total photocurrent measured by the functionalized SiC OSA device can be used to monitor the *I*_0_ signal, we compared the electrical response of the SiC diode with the photon count rate detected by the STXM detector (in this case, the Hamamatsu S12023-05 APD) without any sample present across a set of different energies of the X-ray beam.

**Figure 7:**
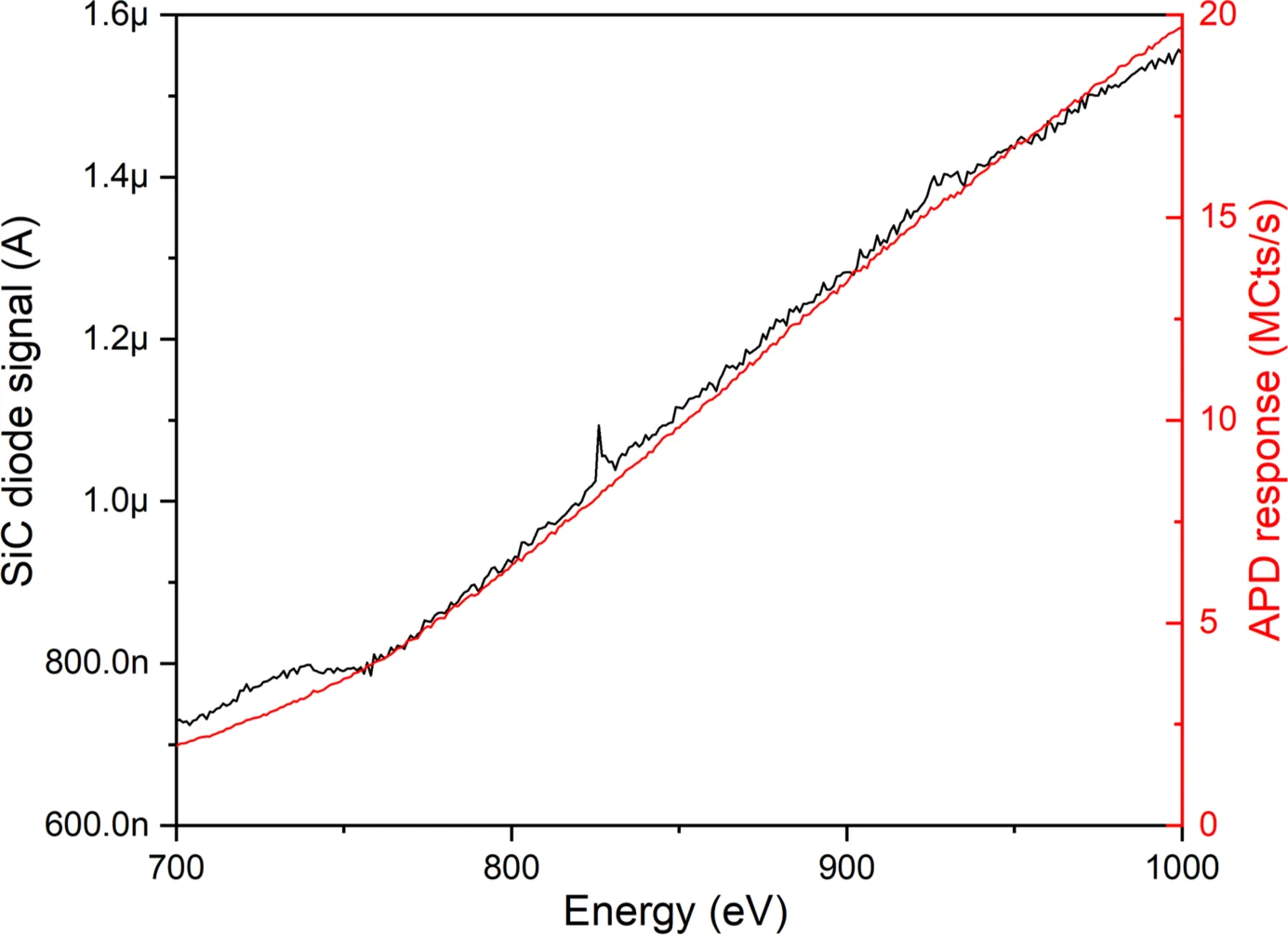
Response comparison between the SiC OSA detector and the APD used for STXM measurements at varying X-ray beam energy, from 700 eV to 1000 eV. A relatively good overlap between the two measurements can be observed.

In particular, the measurements were performed between 700 and 1000 eV in steps of 1 eV with the secondary source slitted down to a 20 µm ×  20 µm square, corresponding to a photon flux delivered to the endstation of about 10^7^ photons s^−1^ (Raabe *et al.*, 2008 ▸). The dwell time for each energy point was 10 ms, comparable to that used for linescan spectroscopy measurements at the PolLux endstation. Fig. 7 ▸ shows the comparison between the recorded SiC detector signal and the reference detector response. The two measurements show a reasonable overlap within the probed energy range, providing a first proof-of-concept that the measurement of the unfocused zeroth-order X-ray beam can be used as an estimation of the *I*_0_ signal on the sample.

A linear correlation analysis was performed between the total photocurrent measured by the three connected channels of the SiC detector and the photon count rate recorded by the reference APD in the 700–1000 eV energy range. A slope of 4.89 (1) nA (MCts s^−1^)^−1^ is determined. The coefficient of determination *R*^2^ of 0.997 indicates that the SiC detector signal follows the intensity variations measured by the reference detector with a good linearity across the full energy interval.

The small deviation between the SiC photocurrent and APD response, in which the SiC detector slightly overestimates the intensity at low energies and slightly underestimates it at higher energies, is not to be attributed to a response variation of the diode but rather to a change in the FZP focusing efficiency, changing the ratio between the intensity in the unfocused 0th order and the one in the focused first order. This highlights the need for an accurate calibration prior to the complete use of such a detector. It is important to notice that this calibration curve will be specific to the beamline optics and FZP utilized for the measurements, *i.e.* it should be repeated following each major change in the endstation/beamline setup. It should also be noticed that a non-negligible measurement noise is still present in the recorded SiC signal. This is likely caused by the noise floor of the current readout chain, *i.e.* trans-impedance amplifier, picoammeter and cabling/EMI pickup. Additional improvements on the electronics will still need to be performed to reduce the measurement noise of the *I*_0_ signal from the SiC diode.

**Figure 8:**
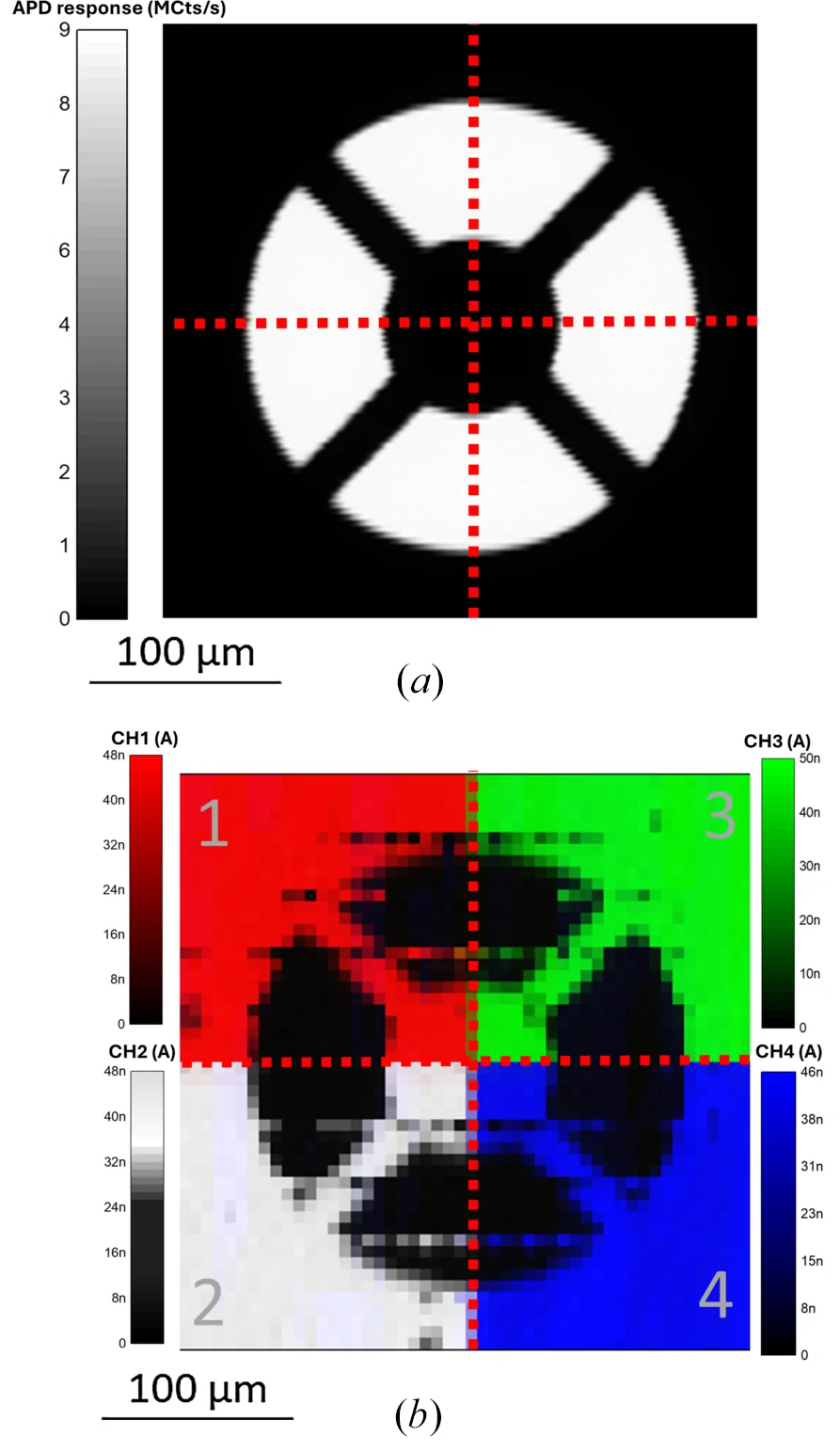
(*a*) STXM image of the X-ray beam illuminating the SiC CS shown in Fig. 4 ▸. (*b*) Response of the four individual sectors of the SiC diode [marked in (*a*)] to the focused X-ray beam illuminating the section of the diode.

Following the proof-of-concept measurements with the functionalized OSA described above, we also investigated the functionalized SiC CS devices. While the CS is normally positioned upstream from the FZP, for the measurements presented here it was positioned downstream of the FZP, in the sample plane, in order to allow us to image it with a focused beam. Fig. 8 ▸(*a*) shows the acquired STXM image and Fig. 8 ▸(*b*) shows the current recorded by the four sectors of the SiC diode.

The main conclusion that can be drawn from the data shown in Fig. 8 ▸(*b*) is that all four sectors exhibit a clear electrical response both in the central and surrounding regions, confirming that the backside PFIB milling of the diode did not electrically isolate the central CS region from the surrounding diode structure. In normal operating conditions (*i.e.* CS positioned upstream from the FZP), the signal from the four sectors of the SiC diode could then be used not only as a complementary diagnostic element for the measurement of the *I*_0_ signal but also for the localization of the beam illuminating the FZP, to correct eventual changes in the beam position occurring during a measurement (*e.g.* by providing a feedback actuation on the first mirror of the beamline).

### 3.2. Discussion

The proof-of-concept measurements presented above demonstrate the possibility of integrating monolithic SiC diode detectors as functionalized optical elements in nanofocus beamlines to perform beam diagnostic measurements. Unlike conventional approaches based on upstream or spatially separated detectors, the functionalized optics provide direct access to beam intensity and position information at locations that are as close as possible to those experienced by the sample, allowing the measured electrical currents from the diode to be used as normalization *I*_0_ signal.

A key outcome of this study is that the integration of apertures and center-stop geometries into active SiC diodes does not compromise the electrical performance of the devices. The PFIB-based fabrication approach enables the precise definition of optical features while preserving diode integrity, which is critical for maintaining linear and stable photocurrent response under soft X-ray illumination. This is particularly relevant for position-sensitive applications, where junction damage or leakage currents would directly degrade spatial resolution and signal fidelity.

The OSA-based devices demonstrate clear and monotonic sector responses to beam displacement, confirming that four-sector SiC diodes provide sufficient sensitivity for transverse beam position reconstruction in the focal plane of an STXM. The present work focuses on qualitative and relative position sensitivity; the observed signal stability and symmetry suggest that quantitative beam position monitoring is feasible. This capability is especially important for long-duration spectroscopic measurements and high-resolution imaging, where slow drifts and vibrations are a dominant source of artifacts. However, this observation cannot be considered as an independent measurement of the focal spot position at the sample plane. According to the thin lens model, the pure displacement of the beam on the zone plate shifts the illuminated part of the pupil but does not shift the focal spot itself, while the incident angular deviation causes focal spot shifting, proportional to the focal length of the zone plate. For the PolLux geometry presented above, the demagnification factor for the beamline is 11/24. At the sample position, the demagnification factor of the combined FZP and beamline optics is of about 1/5000. Therefore, any source-position variation at the upstream position is expected to be significantly suppressed at the focus plane. The observable effect of this upstream position variation is the variation of the illumination of the zone plate and, as a consequence, focused beam intensity. This is exactly what can be observed via the sector-resolved SiC signal. A full separation of the incident beam displacement and tilting would need two longitudinal position-sensitive measurements.

The center-stop-based detectors further extend the versatility of the approach by enabling diagnostics in a configuration complementary to apertures. The successful implementation of backside PFIB fabrication highlights the importance of process design in preserving electrical connectivity in mechanically delicate structures. The demonstrated electrical continuity of the CS support arms confirms that functionalized optics can be engineered even for geometries that are traditionally challenging to integrate with active detectors.

A qualitative equivalence between the SiC-based *I*_0_ measurements and those obtained with standard STXM detectors was shown across a broad soft X-ray energy range. The ability to acquire *I*_0_ and beam position signals in real-time and in parallel with imaging data open up the possibility of pixel-by-pixel correction of intensity fluctuations and beam motion, which is not easily accessible with conventional diagnostics.

Overall, the presented results show the potential of using SiC-based functionalized X-ray optics for integrated beam diagnostics at the soft X-ray energy range.

## 4. Conclusions and outlook

In this work, a possible strategy for *in situ* beam diagnostics in soft X-ray STXM measurements using monolithic patterned silicon carbide detectors is demonstrated. By integrating sensing functionality directly into OSA and CS geometries, it is possible to achieve simultaneous monitoring of the incident beam intensity and transverse position using the zeroth-order unfocused part of the X-ray beam, determining the first-order component through a dedicated calibration curve measurement, which will be dependent on the FZP utilized for focusing the beam. Such calibration curves can be performed as part of the alignment and calibration routines of the experimental endstation.

The devices were fabricated by substrate thinning combined with PFIB milling, enabling precise patterning without compromising the electrical performance of the SiC diodes. Experimental validation at a synchrotron-based STXM beamline provided a first proof-of-concept of the operation of the functionalized optics under soft X-ray conditions.

By eliminating the need for separate upstream diagnostics and enabling beam monitoring directly at critical optical elements, the presented approach could reduce systematic errors associated with upstream diagnostics or reference measurements performed on non-representative regions, improving data quality and artifact correction in soft X-ray microscopy and spectroscopy.

Future works will focus on further reduction of measurement noise, long-term stability studies under user operation, and the extension of this approach to additional beamline configurations. Another future aim will be to assess the extension of the sensitive energy range down to the carbon, nitrogen and oxygen *K*-edges, where the demand for reliable *I*_0_ normalization signals is critical, due to the non-trivial energy dependence of the delivered photon flux at those edges (Watts *et al.*, 2018 ▸).

## Supplementary Material

Sections S1 (`Further details about devices fabrication') including Figs. S1 and S2. . DOI: 10.1107/S1600577526007113/ys5112sup1.pdf
