# Supplementary material for: Monolithic integration of SiC diode detectors into scanning X-ray microscopy optical elements for in situ beam diagnostics
Source: J Synchrotron Radiat. 2026 Aug 5;33(Pt 5):1338–45. doi: 10.1107/S1600577526007113 (PMC13549331; doi:10.1107/S1600577526007113)
Supplement: Supplementary file 1 [file s-33-01338-sup1.pdf]

JOURNAL OF SYNCHROTRON RADIATION

Volume 33 (2026)

Supporting information for article:

Monolithic integration of SiC diode detectors into scanning X-ray microscopy optical elements for *in situ* beam diagnostics

Gabriele Trovato, Simone Finizio, Samuele Moscato, Joerg Raabe, Joakim Reuteler and Massimo Camarda

S1. Further details about devices fabrication

The SiC chip was kept fixed to a glass support using Crystalbond 509 adhesive wax. The thinning was performed by means of mechanical lapping using a cast-iron disk and a Boron Carbide abrasive powder with a grain size of 9 µm. The process was carried out for 2 hours with an applied pressure of 43.6 kPa in order to achieve a substrate thickness below 100 µm whilst maintaining sufficient mechanical stability for handling and integration.

To guarantee the correct alignment of the OSA pinhole with respect to the diode, especially important for multi-sector diodes, a small alignment hole was milled from the frontside of the SiC diode. The diameter of the alignment hole (about 5-10 µm in diameter) was selected to guarantee that the region on the frontside of the SiC diode damaged by the milling would be milled away in the fabrication of the larger diameter OSA pinhole.

Also in the case of SiC CS detectors, small diameter alignment holes were milled from the frontside of the diode prior to the backside milling. As the central region needs to be preserved, some special considerations (Fig. S2) in the fabrication of the alignment holes had to be taken.

As for the previous steps, the electrical functionality of the single devices was verified with electrical transport measurements prior to and following the cut.

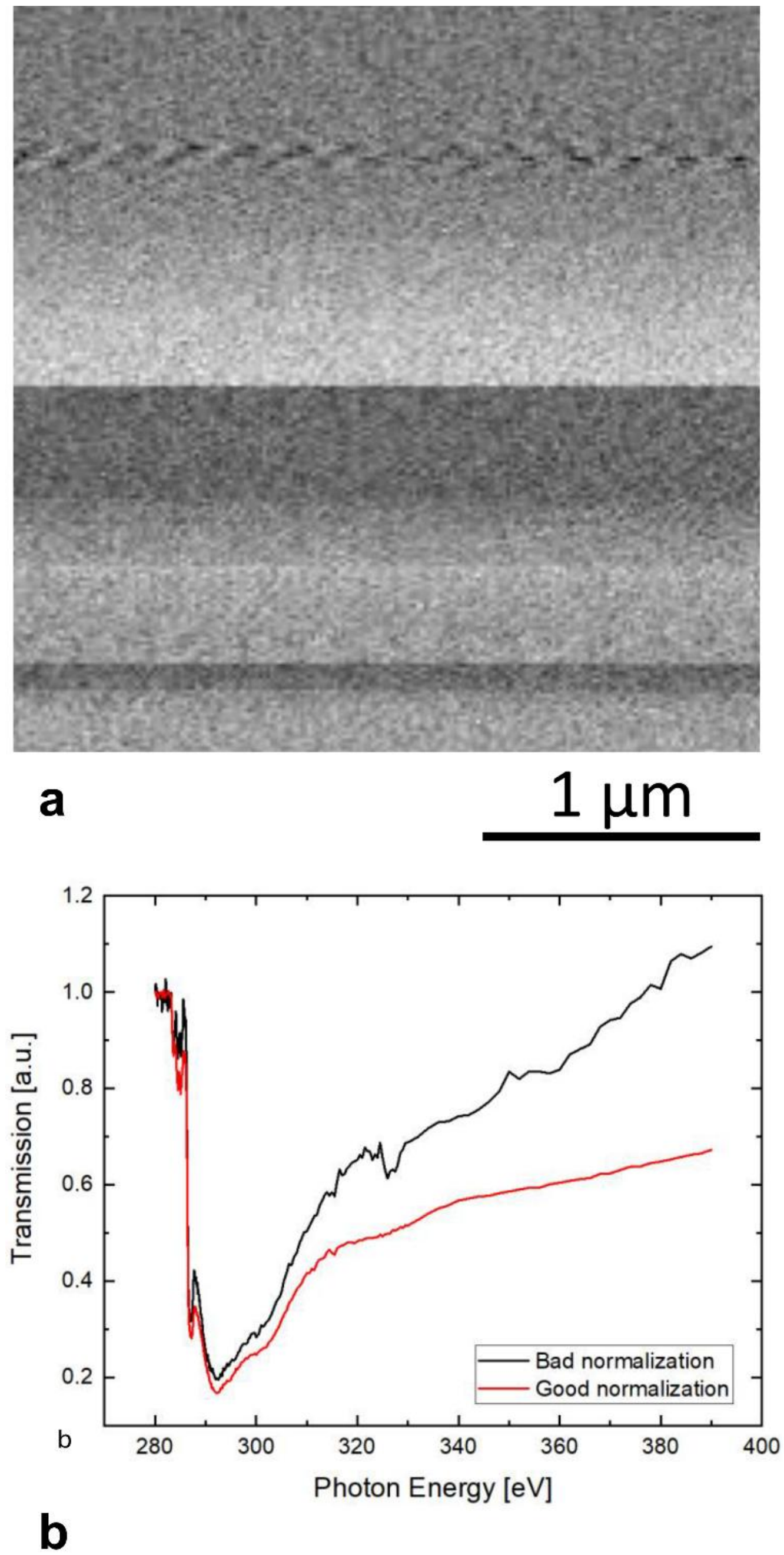

Figure S1 (a) Example showing the typical artifacts arising from vibrations, top-up event, and beam position instabilities. Live monitoring of the beam intensity and position would allow us to correct for these artifacts. (b) Example spectrum at the Carbon K-edge showing the influence of a bad and a good I_0 normalization. Live I_0 beam monitoring would allow for higher quality spectra normalization.

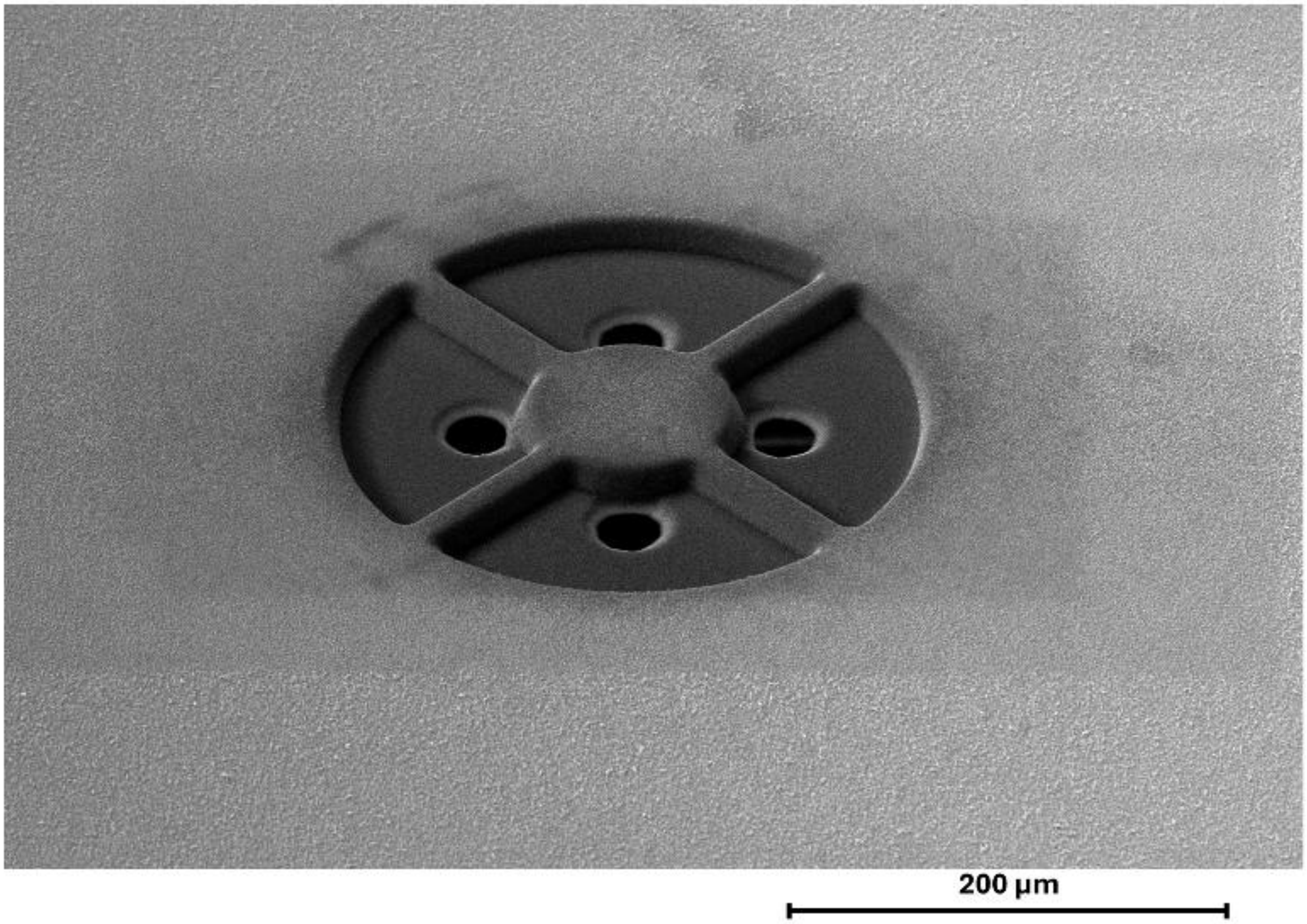

Figure S2 Frontside SEM image of the center stop SiC device during the realization process. The four visible holes are alignment markers necessary to perform the PFIB milling from the backside of the diode.
